# MaveRegistry: a collaboration platform for multiplexed assays of variant effect

**DOI:** 10.1093/bioinformatics/btab215

**Published:** 2021-03-28

**Authors:** Da Kuang, Jochen Weile, Nishka Kishore, Maria Nguyen, Alan F Rubin, Stanley Fields, Douglas M Fowler, Frederick P Roth

**Affiliations:** Donnelly Centre, University of Toronto, Toronto, ON M5S 3E1, Canada; Department of Molecular Genetics, University of Toronto, Toronto, ON M5S 1A8, Canada; Lunenfeld-Tanenbaum Research Institute, Sinai Health, Toronto, ON M5G 1X5, Canada; Department of Computer Science, University of Toronto, Toronto, ON M5T 3A1, Canada; Donnelly Centre, University of Toronto, Toronto, ON M5S 3E1, Canada; Department of Molecular Genetics, University of Toronto, Toronto, ON M5S 1A8, Canada; Lunenfeld-Tanenbaum Research Institute, Sinai Health, Toronto, ON M5G 1X5, Canada; Department of Computer Science, University of Toronto, Toronto, ON M5T 3A1, Canada; Donnelly Centre, University of Toronto, Toronto, ON M5S 3E1, Canada; Department of Molecular Genetics, University of Toronto, Toronto, ON M5S 1A8, Canada; Lunenfeld-Tanenbaum Research Institute, Sinai Health, Toronto, ON M5G 1X5, Canada; Department of Computer Science, University of Toronto, Toronto, ON M5T 3A1, Canada; Donnelly Centre, University of Toronto, Toronto, ON M5S 3E1, Canada; Department of Molecular Genetics, University of Toronto, Toronto, ON M5S 1A8, Canada; Lunenfeld-Tanenbaum Research Institute, Sinai Health, Toronto, ON M5G 1X5, Canada; Department of Computer Science, University of Toronto, Toronto, ON M5T 3A1, Canada; Bioinformatics Division, The Walter and Eliza Hall Institute of Medical Research, Parkville, VIC 3052, Australia; Department of Medical Biology, University of Melbourne, Melbourne, VIC 3010, Australia; Department of Genome Sciences, University of Washington, Seattle, WA 98195, USA; Department of Medicine, University of Washington, Seattle, WA 98195, USA; Department of Genome Sciences, University of Washington, Seattle, WA 98195, USA; Department of Bioengineering, University of Washington, Seattle, WA 98105, USA; Donnelly Centre, University of Toronto, Toronto, ON M5S 3E1, Canada; Department of Molecular Genetics, University of Toronto, Toronto, ON M5S 1A8, Canada; Lunenfeld-Tanenbaum Research Institute, Sinai Health, Toronto, ON M5G 1X5, Canada; Department of Computer Science, University of Toronto, Toronto, ON M5T 3A1, Canada

## Abstract

**Summary:**

Multiplexed assays of variant effect (MAVEs) are capable of experimentally testing all possible single nucleotide or amino acid variants in selected genomic regions, generating ‘variant effect maps’, which provide biochemical insight and functional evidence to enable more rapid and accurate clinical interpretation of human variation. Because the international community applying MAVE approaches is growing rapidly, we developed the online MaveRegistry platform to catalyze collaboration, reduce redundant efforts, allow stakeholders to nominate targets and enable tracking and sharing of progress on ongoing MAVE projects.

**Availability and implementation:**

MaveRegistry service: https://registry.varianteffect.org. MaveRegistry source code: https://github.com/kvnkuang/maveregistry-front-end.

## 1 Introduction

Diagnostic genetic testing frequently yields extremely rare or previously unseen genetic variants, amongst which it can be difficult to identify the subsets of pathogenic and benign variants. Indeed, within the ClinVar repository of clinically observed human variation (Landrum *et al.*, 2018), over 50% of missense variants are classified as ‘variants of uncertain significance’ (VUS) (Weile and Roth, 2018; Starita *et al.*, 2017).

Functional assays provide evidence that can assist variant interpretation (Richards *et al.*, 2015; Brnich *et al.*, 2019). However, such results can arrive weeks or even years after initial discovery of a variant. Multiplexed assays of variant effect (MAVEs) can provide more economical and consistently measured evaluation of variant function than single-variant functional assays (Weile and Roth, 2018; Starita *et al.*, 2017; Brnich *et al.*, 2019). By testing nearly all possible single-nucleotide or amino acid variants in a genomic region of interest, MAVEs can provide ‘proactive’ evidence both for previously observed variants and for variants that will be observed in years to come.

The number of MAVE studies has grown in recent years (Weile and Roth, 2018; Esposito *et al.*, 2019), and includes nearly 100 research labs. Coordination efforts are being launched at the national (e.g. NIH’s Impact of Genomic Variation on Function Consortium) and international (e.g. Atlas of Variant Effects Alliance) levels. To facilitate collaboration and communication at this scale, we created the MaveRegistry. This resource aims to serve diverse stakeholders, including interested researchers, clinicians, patients, patient advocates and funders.

MaveRegistry enables users to: (ii) browse posted MAVE studies; (ii) nominate targets for variant effect mapping; (iii) share progress on ongoing or published MAVE studies; and (iv) follow progress updates from research teams. We expect that the MaveRegistry platform will reduce unintentional competition, promote collaboration and provide efficient communication about systematic variant effect mapping studies, while attracting additional interest from the general public and funding bodies.

## 2 Access projects via a role-based model

**Fig. 1.**
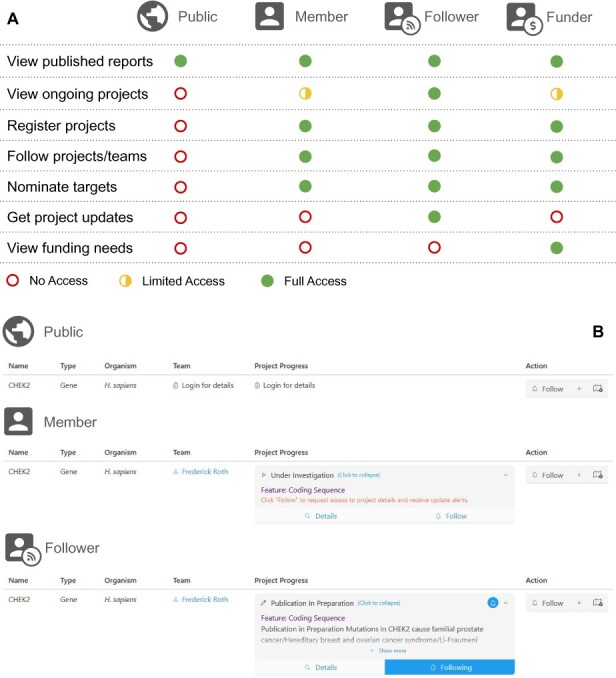
Features of MaveRegistry. (**A**) A role-based model for user-controllable access to information about ongoing MAVE studies. (**B**) Information access depends on user role

‘Real time’ sharing of progress on unpublished projects, which remains rare in academia, can be beneficial (Fischer and Zigmond, 2010). For example, progress-sharing in generating gene-knockout mouse embryonic stem cells (Austin *et al.*, 2004) has helped to inform and avoid duplicated efforts. However, researchers may fear being ‘scooped’ by a competitor (Theologis and Davis, 2004). To encourage progress-sharing for MAVE studies, we designed a role-based model (Fig. 1A) to enable users providing project updates to control who can access their information. These roles are ‘public’, ‘member’, ‘follower’ and ‘funder’.

For MAVE projects marked as ‘published’, anyone can (with a ‘public’ role in Fig. 1A) use MaveRegistry, without logging in, to access all information. Such records include a brief project summary and links to publications and data.

Users can create an account and thus become ‘members’ (see Fig. 1A). Member researchers can deposit their ongoing or published MAVE projects. Any member can request permission to view or even edit posted MAVE projects. If approved by the depositor to ‘follow’ a project, members (now with a ‘follower’ role in Fig. 1A) can view project details. Members may also request permission to follow research teams. Followers of either projects or teams will receive notifications about status changes in relevant projects. Members affiliated with funding agencies may take a ‘funder’ role (Fig. 1A), granting access to the (optionally disclosed by depositors) funding needs for each MAVE project. Figure 1B exemplifies the information visible to users with different roles.

## 3 Register and manage MAVE projects

Members can deposit ongoing or published variant effect mapping projects. If a genomic region (i.e. target element) has not been previously submitted, the submitter will be prompted for basic information, including target name, type (i.e. protein-coding gene or other genomic region) and relevant organism. The depositor then provides contact information for a project lead, to be shared with other members to facilitate direct communication. A research team is named for each project, enabling a ‘view projects by research team’ function. To describe a project’s status, depositors can select terms from a controlled vocabulary of project activity. For each activity, the start and (if appropriate) end date can be entered. A brief free-text progress summary, aided by template questions, can also be provided. Depositors can also provide links to external sources, e.g. relevant publications, code repositories, online lab notebooks or protocol resources.

When members make ‘follow’ requests, the relevant depositors are notified. Depositors can review the reason-to-follow message, approve or reject each follow request, provide edit access (e.g. to collaborators), remove followers or transfer ownership of their projects to other members.

## 4 Nominate targets for variant effect mapping

Diverse stakeholders, e.g. patients, patient advocates, clinicians or researchers with an interest in the fundamentals of sequence-structure relationships, may wish to nominate new targets for MAVE studies. MaveRegistry therefore provides a nomination interface, and enables other members to ‘upvote’ or ‘downvote’ nominations. When the nominated target is a human protein-coding gene, users may link to MaveQuest, an online resource for identifying potential functional assays and other information about clinical relevance (Kuang *et al.*, 2020).
